# Genome-Wide Identification and Homoeologous Expression Analysis of *PP2C* Genes in Wheat (*Triticum aestivum* L.)

**DOI:** 10.3389/fgene.2019.00561

**Published:** 2019-06-12

**Authors:** Xiaofen Yu, Jiapeng Han, Efan Wang, Jie Xiao, Rui Hu, Guangxiao Yang, Guangyuan He

**Affiliations:** The Genetic Engineering International Cooperation Base of Chinese Ministry of Science and Technology, Key Laboratory of Molecular Biophysics of Chinese Ministry of Education, College of Life Science and Technology, Huazhong University of Science and Technology, Wuhan, China

**Keywords:** wheat, protein phosphatase 2C (PP2C), genome-wide, gene expression, homoeologous pattern, stress response

## Abstract

Plant protein phosphatase 2Cs (PP2Cs) play crucial roles in phytohormone signaling, developmental processes, and both biotic and abiotic stress responses. However, little research has been conducted on the *PP2C* gene family in hexaploid wheat (*Triticum aestivum* L.), which is an important cereal crop. In this study, a genome-wide investigation of *TaPP2C* gene family was performed. A total of 257 homoeologs of 95 *TaPP2C* genes were identified, of which 80% of genes had all the three homoeologs across A, B, and D subgenomes. Domain analysis indicated that all the *TaPP2C* homoeologs harbored the type 2C phosphatase domains. Based on the phylogenetic analysis, TaPP2Cs were divided into 13 groups (A-M) and 4 single branches, which corresponded to the results of gene structure and protein motif analyses. Results of chromosomal location and synteny relationship analysis of *TaPP2C* homoeologs revealed that known chromosome translocation events and pericentromeric inversions were responsible for the formation of *TaPP2C* gene family. Expression patterns of *TaPP2C* homoeologs in various tissues and under diverse stress conditions were analyzed using publicly available RNA-seq data. The results suggested that *TaPP2C* genes regulate wheat developmental processes and stress responses. Homoeologous expression patterns of *TaPP2C* triad homoeologs from A, B, and D subgenomes, revealed expression bias within triads under the normal condition, and variability in expression under different stress treatments. Quantitative real-time PCR (qRT-PCR) analysis of eight *TaPP2C* genes in group A revealed that they were all up-regulated after abscisic acid treatment. Some genes in group A also responded to other phytohormones such as methyl jasmonate and gibberellin. Yeast two-hybrid assays showed that group A TaPP2Cs also interacted with TaSnRK2.1 and TaSnRK2.2 from subclass II, besides with subclass III TaSnRK2s. *TaPP2C135* in group A was transformed into *Arabidopsis* and germination assay revealed that ectopic expression of *TaPP2C135* in *Arabidopsis* enhanced its tolerance to ABA. Overall, these results enhance our understanding of the function of TaPP2Cs in wheat, and provide novel insights into the roles of group A TaPP2Cs. This information will be useful for in-depth functional analysis of TaPP2Cs in future studies and for wheat breeding.

## Introduction

Reversible protein phosphorylation and dephosphorylation by protein kinases and protein phosphatases (PPs), respectively, are essential for the regulation of various biological processes in plants. Depending on substrate specificity, PPs are classified into three major categories: tyrosine phosphatases (PTPs), serine/threonine phosphatases (PSPs), and dual-specificity phosphatases (DSPTPs). Based on amino acid sequences and crystal structures, PPs are divided into two families: the Mg^2+^/Mn^2+^- dependent protein phosphatase (PPM) family and the phosphor-protein phosphatase (PPP) family. The PPP proteins harbor both catalytic and regulatory subunits, whereas PPM proteins carry only catalytic subunits (Shi, 2009). Type 2C protein phosphatase (PP2C) is a kind of PSP which requires Mg^2+^ for its activity; therefore, PP2C also belongs to the PPM family (Luan, 1998; Schweighofer et al., 2004). Plant PP2Cs are involved in various signaling cascades including phytohormone signaling networks like abscisic acid (ABA), salicylic acid (SA)-ABA crosstalk, and developmental processes like mitogen-activated protein kinase (MAPK) signaling, and CLAVATA (CLV) signaling pathway (Song et al., 2006; Ma et al., 2009; Umbrasaite et al., 2010; Manohar et al., 2017).

In *Arabidopsis thaliana*, 80 *PP2C* genes have been identified. Of these 80 AtPP2C proteins, 73 are grouped into 12 subfamilies (A-L), while seven are ungrouped (Xue et al., 2008). The AtPP2C proteins in group A have been well-studied as ABA co-receptors, which negatively regulate the ABA signaling pathway. All *AtPP2C* genes in group A have been identified in *Arabidopsis*, including *ABA-INSENSTIVE1* (*ABI1*) and *ABI2* (Leung et al., 1994, 1997). Under normal conditions, group A AtPP2Cs bind to subclass III SNF1-related protein kinases (SnRK2s), thus inactivating SnRK2s (Yoshida R. et al., 2006; Umezawa et al., 2009), while under abiotic stresses, PYR/PYL/RCARs coupling ABA combine with group A AtPP2Cs to release SnRK2s, thus activating downstream factors such as ABA responsive element (ABRE)-binding factors (ABFs) to respond to the environmental stress (Ma et al., 2009; Park et al., 2009; Umezawa et al., 2010). Similarly, in hexaploid wheat (*Triticum aestivum* L.), TaABI1 binds to subclass III TaSnRK2s (Zhang et al., 2016), and Ta_PYL2DS_FL inhibits TaABI1 proteins in an ABA-dependent manner *in vitro* (Gordon et al., 2016). Recent research showed that ABFs bound to the promoters of group A *PP2C* genes *in vivo*, this binding was further promoted by ABA treatments. Thus, group A PP2Cs and ABFs regulated homeostasis of ABA signaling *via* a feedback loop (Wang X. et al., 2018). Additionally, ABA induces the degradation of ABI1 *in vivo* by the 26S proteasome to enhance ABA signaling (Kong et al., 2015). Some PP2Cs in other groups have also been characterized. Four members (AP2C1-4) of group B AtPP2Cs function as mitogen-activated protein kinase (MAPK) phosphatases. In *Arabidopsis*, AP2C1 regulates phytohormone and defense responses by inactivating MPK4 and MPK6 (Schweighofer et al., 2007), whereas AP2C3 mediates stomata development, thus negatively regulating MAPK signaling (Umbrasaite et al., 2010). Among group C PP2Cs, POLTERGEIST (POL) and PLL1 mediate CLV1 signaling and are essential for stem-cell maintenance and differentiation (Yu et al., 2003; Song et al., 2006). Group D comprises nine PP2Cs in *Arabidopsis*, all of which exhibit different subcellular localization and expression patterns (Tovar-Mendez et al., 2014). Additionally, *AtPP2CD5, D6, D7*, and *D9* exhibit diverse responses to alkali stress (Chen et al., 2017). Moreover, AtPP2CD2, D5, and D6 proteins negatively regulate SAUR-mediated cell expansion (Ren et al., 2018). In group E, AtPP2C-6-6 inactivates histone acetyltransferase GCN5 by dephosphorylation (Servet et al., 2008). Overexpression of another group E *PP2C* gene, *AtPP2CF1*, increases plant biomass in *Arabidopsis* (Sugimoto et al., 2014). *WIN2*, a group F *PP2C* gene, is required for bacterial effector HopW1-1-induced disease response in *Arabidopsis* (Lee et al., 2008). *AtPP2CG1*, a group G *PP2C* gene is a positive regulator of salt tolerance in an ABA-dependent manner (Liu et al., 2012). AtPP2C62 and AtPP2C26, which belong to group K, are involved in the suppression of plant immune response and pathogen resistance (Akimoto-Tomiyama et al., 2018). An unclassified PP2C protein KAPP, interacts with several receptor-like protein kinases (RLKs) such as CLV1, and therefore is involved in CLV1 signaling (Stone et al., 1994; Trotochaud et al., 1999). Nevertheless, apart from the above mentioned groups, functions of PP2Cs in other groups in *Arabidopsis* have not yet been elucidated. Moreover, fewer studies on the *PP2C* gene family have been conducted in monocots. In rice (*Oryza sativa*) and *Brachypodium distachyon*, 78 and 86 *PP2C* genes have been identified by computational analysis, respectively (Xue et al., 2008; Cao et al., 2016). However, little is known about the functions of PP2Cs in hexaploid wheat.

Wheat is one of the three main staple food crops, and the most widely cultivated crop around the world. Common wheat is allohexaploid (AABBDD) with a large and complex genome (approximately 17 GB), more than 85% of which is repetitive DNA (International Wheat Genome Sequencing Consortium [IWGSC], 2014). Present-day hexaploid wheat was formed *via* polyploidization through twice interspecific hybridization events. Tetraploid wheat (*Triticum turgidum*; AABB) was derived from hybridization between wild diploid wheat (*Triticum urartu*; A subgenome donor) and a close relative of *Aegilops speltoides* (B subgenome donor). Hexaploid wheat (AABBDD) originated from hybridization between tetraploid wheat and diploid *Aegilops tauschii* (D subgenome donor). Most homeologous chromosomes are collinear (Dvorak and Akhunov, 2005), except chromosomes 4A and 5A, which underwent reciprocal translocations combined with pericentromeric inversions between chromosome arms. Three additional known translocation events include 7BS-4AL (Devos et al., 1995), 5AL-7BS (Ma et al., 2013), and 5BS-4BL (Devos et al., 1993). In August 2018, the first fully annotated reference genome of hexaploid wheat was completed by the International Wheat Genome Sequencing Consortium (IWGSC), making it more convenient and efficient to analyze gene functions in wheat, thus accelerating wheat research (Appels et al., 2018).

In this study, we performed a genome-wide investigation of the *PP2C* gene family in hexaploid wheat. A total of 257 *TaPP2C* gene homoeologs were identified and were divided into 13 groups by phylogenetic analysis. Chromosomal distribution, duplication event, exon-intron structure and protein motif of these *TaPP2C* genes were also analyzed. Subsequently, expression patterns of *TaPP2C* homoeologs in different tissues and under various stress conditions were analyzed *in silico*. Additionally, the expression profiles of eight *TaPP2C* genes from group A were analyzed after drought, salt, ABA, and other phytohormone treatments by quantitative real-time PCR (qRT-PCR). Yeast two-hybrid assay was performed to validate interactions between group A TaPP2Cs and TaSnRK2s. Finally, *TaPP2C135* in group A was transformed into *Arabidopsis* for further functional analysis.

## Materials and Methods

### Identification and Phylogenetic Analysis

To identify the *TaPP2C* genes in hexaploid wheat, amino acid sequences of all PP2Cs in *Arabidopsis* and rice were downloaded from The Arabidopsis Information Resource (TAIR^1^) and Rice Genome Annotation Project (RGAP^2^) databases with the National Center for Biotechnology Information (NCBI^3^) as a complementary database. These sequences were then used as queries to perform BLASTp and tBLASTn searches (threshold *e*-value < 1e-10) against the *T. aestivum* reference sequences in the Ensembl Plants database^4^ supported by the IWGSC database^5^. Then, the candidate *TaPP2C* genes were used as queries to perform BLASTn searches of the wheat genome to obtain more potential genes. The hmmsearch program of the HMMER software^6^ (version 3.2.1) was also applied to the identification of TaPP2Cs using protein phosphatase 2C domain (PF00481, PF07830, and PF13672) in Pfam 32.0 database^7^. The above obtained protein sequences were further screened for conserved domains using SMART^8^ and NCBI Conserved Domains with automatic mode (threshold = 0.01; maximum number of hits = 500)^9^, and proteins without a typical PP2C catalytic domain were removed. Multiple sequence alignment of TaPP2C amino acid sequences was performed using ClustalX 2.1 then a phylogenetic tree was generated using MEGA 6.0 based on the neighbor-joining (NJ) method with 1000 bootstrap replicates (Larkin et al., 2007; Tamura et al., 2011). Nonsynonymous (Ka) and synonymous (Ks) substitution rates were calculated by Ka/Ks Calculator 2.0^10^ using the Nei and Gojobori (NG) method (Wang et al., 2010).

### Sequence Analysis

To map all *TaPP2C* genes to wheat chromosomes, the genome annotation file IWGSC RefSeq v1.0 was downloaded from the IWGSC database. Multiple sequence alignment of *TaPP2C*s was performed to analyze gene duplication events among the three subgenomes (A, B, and D) of hexaploid wheat. Subsequently, synteny blocks of *TaPP2C*s were calculated using MCScanX with *e*-value ≤ 1e-10 (Wang et al., 2012). Chromosomal locations and syntenic relationships were illustrated using Circos-0.67. To analyze the chromosomal translocation events in the wheat genome, data provided by Ma et al. (2013) and Clavijo et al. (2016) were used. The coding sequences and genome sequences of *TaPP2C*s were used to determine the exon-intron structures by Gene Structure Display Server^11^ (Hu B. et al., 2015). To identify conserved motifs within TaPP2Cs, the MEME motif search tool (Bailey et al., 2009) was applied with an optimum motif width of 6–50 and each motif having 2–600 sites. The results were rearranged by TB tools.

### Expression Pattern Analysis

To analyze the expression patterns of *TaPP2C*s, RNA-seq data of the project choulet_URGI (Ramírez-González et al., 2018), DRP000768 (Oono et al., 2013), SRP041017 (Zhang et al., 2014), SRP043554 (Li et al., 2015), and SRP045409 (Liu et al., 2015) were downloaded from the expVIP platform^12^. Heatmaps were generated from log 2 based transcripts per million (TPM) values using pheatmap package of R project^13^. Expression data of those *TaPP2C*s represented in all the three homoeologous subgenomes were chosen to analyze the homoeologous expression patterns using SigmaPlot^14^.

### Plant Materials and Treatments

The hexaploid wheat (*T. aestivum* L. cv. Chinese Spring) seeds were surface sterilized, then were soaked in distilled water in a greenhouse (16 h light/8 h dark cycle at 22°C). After 2 weeks, young seedlings were steeped in and sprayed with 200 mM NaCl, 20% (w/v) polyethylene glycol (PEG) 6000, 100 μM ABA, 100 μM gibberellin (GA) and 100 μM methyl jasmonate (MeJA) for 24 h, respectively. The seedlings treated with distilled water were used as the controls. The leaf tissues from seedlings were harvested at six different time points (0, 1, 3, 6, 12, and 24 h) after treatments. All leaf samples, including treated and control samples, were collected with three biological replicates at each time point, and were stored at -80°C till the extraction of total RNA.

### Expression Analysis by qRT-PCR

Total RNA was extracted from each sample using a Plant Total RNA extraction Kit (Zomanbio, Beijing, China), according to the manufacturer’s instruction, and stored at -80°C. First-strand cDNA was synthesized from total RNA (50 ng–2 μg) in a 20 μl volume using FastKing RT Kit (Tiangen, Beijing, China), according to the instructions. The concentration of total RNA varied from 0.01 to 1 μg/μl. Next, qRT-PCR was performed on a real-time PCR instrument (CFX96; Bio-Rad, Hercules, CA, United States) using AceQ qPCR SYBR Green Master Mix (Vazyme, Nanjing, China). To identify *cis*-regulatory elements in gene promoters, approximately 2 kb upstream sequences of genes were analyzed *via* PlantCARE search tool^15^.

### Yeast Two-Hybrid Assays

A total of six *TaPP2C*s in group A and ten *TaSnRK2*s were amplified from the wheat cDNA. The *TaPP2C* and *TaSnRK2* genes were cloned into pGADT7 and pGBKT7 vectors, respectively. Primers for the amplification of *TaSnRK2*s were obtained from Zhang et al. (2016). Yeast two-hybrid assay was performed according to the manufacturer’s protocol (Clontech, United States) using yeast strain AH109. Positive transformants picked from SD medium lacking leucine and tryptophan (SD/-Leu/-Trp) were subsequently transferred to auxotrophic medium for further selection.

### Transformation of *Arabidopsis*

The pSN1301-*TaPP2C135* plasmid and pSN1301 empty vector were transformed into *Arabidopsis* using the floral-dip method with *Agrobacterium tumefaciens* strain EHA105 (Clough and Bent, 1998). Seeds of transgenic *Arabidopsis* were selected using Murashige and Skoog (MS) medium (pH 5.8) supplemented with 20 mg/L hygromycin B. Homozygous lines of T_3_ and T_4_ generations were used for germination analysis. For germination assay, approximately 50–60 seeds were sown on MS plates containing various concentrations of ABA. After stratification for 4 days, the germination greening ratio was scored daily for consecutive 7 days.

## Results

### Genome-Wide Identification and Characterization of *TaPP2C* Genes

**FIGURE 1:**
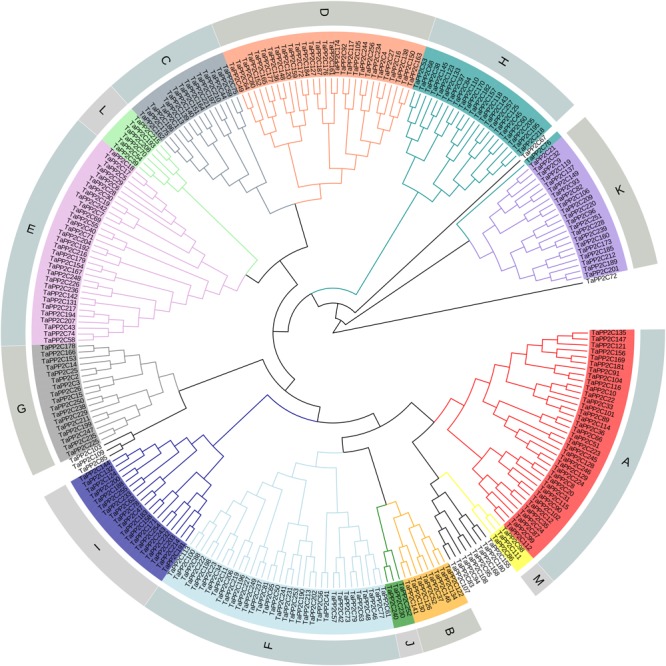
Phylogenetic analysis of TaPP2C proteins. A total of 257 TaPP2C proteins were used to construct the phylogenetic tree using neighbor-joining method with ClustalX 2.1 and MEGA 6.0 with 1,000 bootstrap replicates. The PP2C proteins are divided into 13 distinct groups (A–M), which are indicated with different colors except for the ungrouped PP2C proteins.

After the genome-wide searching and characterization of PP2C catalytic domain, a total of 257 *PP2C* homoeologs in wheat were identified (Supplementary Table S1). These *TaPP2C* genes were renamed based on the order of wheat subgenomes (A, followed by B and D), chromosomes (1–7), and positions on each chromosome. Phylogenetic analysis of TaPP2Cs and OsPP2Cs was performed to analyze the evolutionary relationships (Supplementary Figure S1). An individual phylogenetic tree of the TaPP2C proteins was also made to separately check their phylogenetic relationships (Figure 1). The result indicated that these TaPP2C proteins could be divided into 13 groups (A-M) with 11 ungrouped proteins, which was consistent with the PP2C groups found in rice and *Arabidopsis*.

Hexaploid wheat contains three (A, B, and D) homoeologous subgenomes. Consequently, every hexaploid wheat gene potentially owns three homoeologs from three homoeologous chromosomes as a triad. Blast searches against the wheat genome revealed that the 257 *TaPP2C* genes represented 257 homoeologs of 95 genes: 76 genes had all the three homoeologs, ten genes had two homoeologs, nine genes had only one homoeolog (Table 1 and Supplementary Table S2). The distribution of *TaPP2C*s in each group was similar to that of *AtPP2C*s and *OsPP2C*s, except for groups A, H, and K: these groups contained more members in wheat than in *Arabidopsis* and rice (Table 2).

**Table 1:** The subgenome distribution of *TaPP2C* homoeologs.

Distribution pattern		Number of genes
Three homoeologs	A, B, D	76
Two homoeologs	A, B	4
	A, D	3
	B, D	3
One homoeolog	A	3
	B	3
	D	3
Total homoeologs	257	95

**Table 2:** The distribution of PP2Cs in wheat, *Arabidopsis* and rice.

Group of PP2C	Number of AtPP2Cs	Number of OsPP2Cs	Number of TaPP2Cs
A	9	10	15
B	6	3	3
C	7	5	6
D	9	10	10
E	12	8	12
F	12	12	12
G	6	5	6
H	3	5	8
I	2	11	7
J	2	1	1
K	3	4	9
L	2	1	2
M	0	1	1
Single Branch	7	2	4
Total Number	80	78	95

### Analysis of Chromosomal Location and Duplication of *TaPP2C* Genes

**FIGURE 2:**
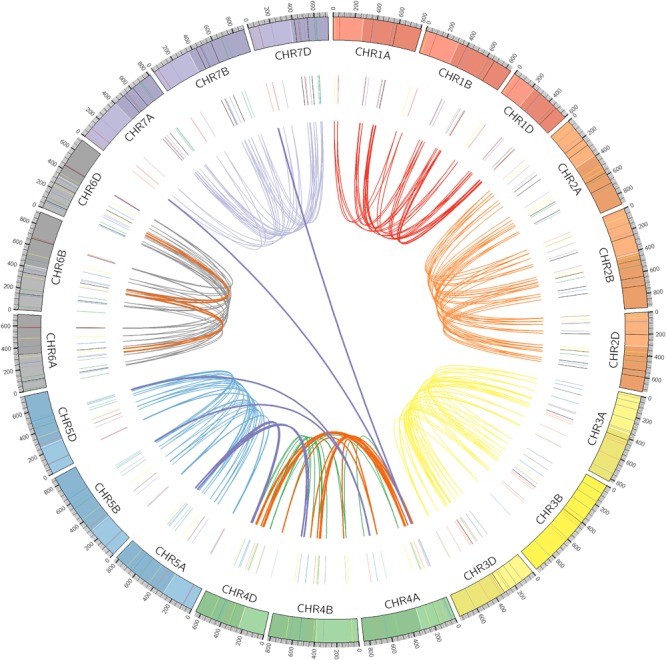
Chromosomal distribution and duplication events of *TaPP2C* genes. All *TaPP2C* homoeologs were mapped to 21 wheat chromosomes (7 chromosomes of the A, B, and D subgenomes) using Circos. Chromosome number is indicated inside the outer circle. Different groups of *TaPP2C* genes are highlighted with different colored lines inside. Links in the central circle connect *TaPP2C* homoeolog triads. Bold orange links and bold purple links connect homoeologs involved in pericentromeric inversions and translocation events, respectively.

Chromosomal locations as well as syntenic relationships among the A, B, and D subgenomes of *TaPP2C* genes are illustrated in Figure 2. All *TaPP2C* homoeologs were mapped to 21 wheat chromosomes, which were highlighted in the middle circle in Figure 2. The *TaPP2C* genes in different groups (indicated in different colors in Figure 2) showed an uneven distribution across the A, B, and D subgenomes and unbiased distribution among the seven chromosomes of each subgenome. *TaPP2C* homoeologs involved in chromosome translocation and pericentromeric inversion events were identified (Table 3), and these crosslinks were also represented in the inner circle of Figure 2. Six triads (18 homoeologs) were involved in pericentromeric inversions between the long and short arms of chromosome 4A; three triads (nine homoeologs) were involved in reciprocal translocations between the long arms of chromosomes 4A and 5A; and one triad (three homoeologs) was involved in translocation of 7BS and 4AL. Additionally, it was worth mentioning that synteny analysis on *TaPP2C194*/*207*/*217* and *TaPP2C195*/*205*/*218* suggested pericentromeric inversion between the long and short arms of chromosome 6B.

**Table 3:** *TaPP2C* homoeologs involved in chromosome pericentromeric inversions and translocations.

Homoeolog	Location	Event	Homoeolog	Location	Event
*TaPP2C119*	4AS	4AL- > 4AS	*TaPP2C163*	5AL	4AL- > 5AL
*TaPP2C137*	4BL		*TaPP2C138*	4BL	
*TaPP2C149*	4DL		*TaPP2C150*	4DL	
*TaPP2C121*	4AS	4AL- > 4AS	*TaPP2C164*	5AL	4AL- > 5AL
*TaPP2C135*	4BL		*TaPP2C139*	4BL	
*TaPP2C147*	4DL		*TaPP2C151*	4DL	
*TaPP2C122*	4AS	4AL- > 4AS	*TaPP2C127*	4AL	5AL- > 4AL
*TaPP2C134*	4BL		*TaPP2C176*	5BL	
*TaPP2C146*	4DL		*TaPP2C187*	5DL	
*TaPP2C123*	4AS	4AL- > 4AS	*TaPP2C224*	7AS	7BS- > 4AL
*TaPP2C133*	4BL		*TaPP2C129*	4AL	
*TaPP2C145*	4DL		*TaPP2C246*	7DS	
*TaPP2C124*	4AS	4AL- > 4AS	*TaPP2C194*	6AS	6BS- > 6BL
*TaPP2C132*	4BL		*TaPP2C207*	6BL	
*TaPP2C144*	4DL		*TaPP2C217*	6DS	
*TaPP2C126*	4AL	4AS- > 4AL	*TaPP2C195*	6AL	6BL- > 6BS
*TaPP2C130*	4BS		*TaPP2C205*	6BS	
*TaPP2C141*	4DS		*TaPP2C218*	6DL	

To determine the mode of selection of duplicated *TaPP2C* genes in groups A-I and K, Ka/Ks ratios were calculated for each gene-pair (Supplementary Table S3). All of the computed gene pairs showed a Ka/Ks ratio < 1, suggesting that *TaPP2C* genes in these groups underwent purification or negative selection. The Ka/Ks value of three gene pairs (*TaPP2C48*/*63, TaPP2C202*/*213*, and *TaPP2C42*/*57*) was zero, indicating strong purifying selection. The average Ka/Ks ratios of gene pairs in different groups ranged from 0.0808 (group G) to 0.3258 (group I), while the Ka/Ks ratios of genes in group F varied from 0 to 0.756.

### Gene Structure, Protein Domain, and Motif Analysis

**FIGURE 3:**
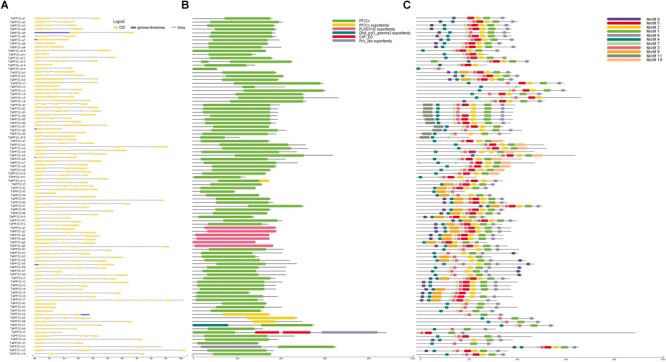
Gene structure, protein domain and motif analysis of TaPP2Cs. **(A)** Exon–intron structures of *TaPP2C* genes. **(B)** Distribution of conserved domains within TaPP2C proteins. **(C)** Distribution of all motifs identified by MEME.

Exon–intron structural diversity within a gene family is an important clue for the evolutionary and functional analyses of gene family members. To examine the structural features of *TaPP2C* genes, one homoeolog of each *TaPP2C* gene was selected and exon-intron structure was analyzed (Figure 3A). The results revealed that genes in the same group shared a similar number of exons but with different exon and intron lengths. A few exceptions were noted in most groups. In group D, while most genes harbored four or five exons, *TaPP2C-d8* and *TaPP2C-d10* contained only three and two exons, respectively. Genes in group K exhibited wide variation in exon number ranging from one to 12, and *TaPP2C-k1, -k2*, and *-k3* genes contained only one exon. We also examined protein domains and conserved motifs in amino acid sequences of TaPP2Cs. Protein domain analysis showed that most members contained typical PP2C catalytic domains, whereas all six members of group G contained PLN03145, which also belonged to PP2C protein family (Figure 3B).

To identify common motifs among different groups of TaPP2C proteins, we used the MEME motif search tool. Eleven conserved motifs were identified (Table 4); the distribution of these motifs in TaPP2C proteins is illustrated in Figure 3C. The motifs identified in TaPP2C proteins resembled those identified in OsPP2Cs and AtPP2Cs, except motifs 9 and 10. Proteins in the same group exhibited similar motif distribution patterns (Figure 3C). Some motifs (1, 3, 4, 6, and 7) were present in most groups, whereas other motifs were specific to one or two groups; for example motifs 10 and 11 were observed only in groups E and D, respectively. Motif 4 containing [DG]X_2_[G](D, aspartic acid; G, glycine; X, any amino acid), is a PPM-type phosphatase signature.

**Table 4:** Conserved motifs in the amino acid sequences of TaPP2C proteins.

Motif	Width	Multilevel consensus sequence
1	29	JTEDDEFLILASDGLWDVLSNZEAVDIVR
2	21	WRVKGGLAVSRAIGDRYLKPY
3	15	LVVANVGDSRAVLSR
4	15	SFFGVFDGHGGPEAA
5	29	GKAVQLSVDHKPBRPDERERIEAAGGRVF
6	15	RGSKDBITVVVVDLK
7	11	GSTAVVAVIVG
8	41	EYLKEHLFENJJKHPKFPTDTKKAISEAYTKTDSDFLESES
9	15	YGCSSCQGRRAEMED
10	40	TPSREKAAKALVECAVRAWRTKYPTSKVDDCAAVCLFLHT
11	40	AAGAQDGLLWYRDLGQHAAGEFSMAVVQANELLEDQSQVE

### Tissue-Specific Expression Profiles of *TaPP2C* Genes

**FIGURE 4:**
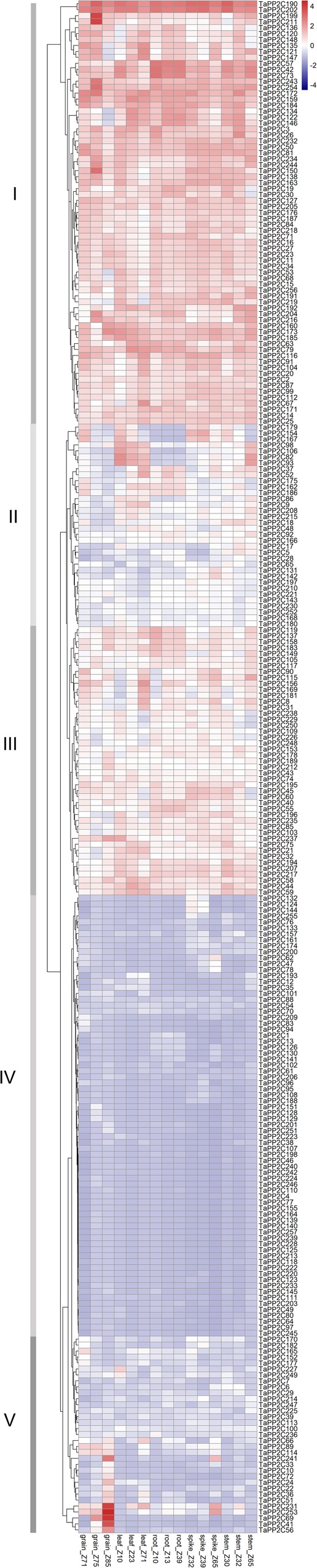
Expression patterns of *TaPP2C* genes in various wheat tissues. Heatmap of *TaPP2C* RNA-seq data in five tissues at three different developmental stages was created by R program. Row clustering was applied. Grain_Z71, _Z75 and _Z85: grains at 2 days post anthesis (dpa), 15, and 30 dpa stages, respectively; Leaf_Z10, _Z23, and _Z71: seedling stage leaf and flag leaf at tillering, 2 dpa stages; Root_Z10, _Z13, and _Z39: roots at seedling, three leaf and flag leaf stage, respectively; Spike_Z32, _Z39, and _Z65: spikes at two-node, flag leaf and anthesis stages, respectively; Stem_Z30, _Z32, and _Z65: stems at 1 cm spike, two-node and anthesis stages, respectively. Red and blue cells indicate relative higher or lower expression.

To clarify the biological roles of *TaPP2C* genes in wheat, expression patterns of all *TaPP2C* homoeologs were analyzed. RNA-seq data across 15 tissues (five tissues at three different developmental stages) of the wheat cultivar Chinese Spring under non-stress conditions were used to analyze the spatial and temporal expression patterns of *TaPP2C* genes (Figure 4). There were four homoeologs with no detectable expression, which might merely express at other specific tissues or under special conditions. Most *TaPP2C* genes exhibited a broad range of expression in stem, spike, root, leaf and grain tissues of wheat plants at different developmental stages.

Row clustering was applied, and as a result, the 257 homoeologous *TaPP2C* genes fell into five groups (I-V) based on expression patterns. Genes in groups I and IV, which accounted for nearly two-thirds of the *TaPP2C* genes, maintained similar expression levels in all 15 tissues. Additionally, expression levels of genes in groups I and IV were higher or lower, respectively, than those in other groups. Most genes in groups II and III exhibited moderate expression levels, although the expression level varied at different developmental stages. Expression levels of group II genes including *TaPP2C82, 93*, and *106* were dramatically higher in leaf and stem tissues than in other tissues at all developmental stages. Notably, *TaPP2C82, 93*, and *106* identified as homoeologs of the same gene in group K, displayed parallel expression pattern. However, not all homoeologs of the same gene showed similar expression patterns, *TaPP2C86, 98*, and *111*, homoeologs of gene *TaPP2C-m1*, showed diverse expression patterns in groups II and IV respectively, suggesting functional diversification of homoeologous genes. Expression levels of genes in group V were relative low in most tissues with the exception of particular one or two tissues. Group V contained 20 homoeologs, all of which showed highly preferential expression in grain; *TaPP2C41, 56*, and *69* showed grain-specific expression especially at 30 days post anthesis. However, no obvious comparable expression pattern was observed within different groups of *TaPP2C* genes.

### Expression Patterns of *TaPP2C* Genes Under Various Stress Conditions

**FIGURE 5:**
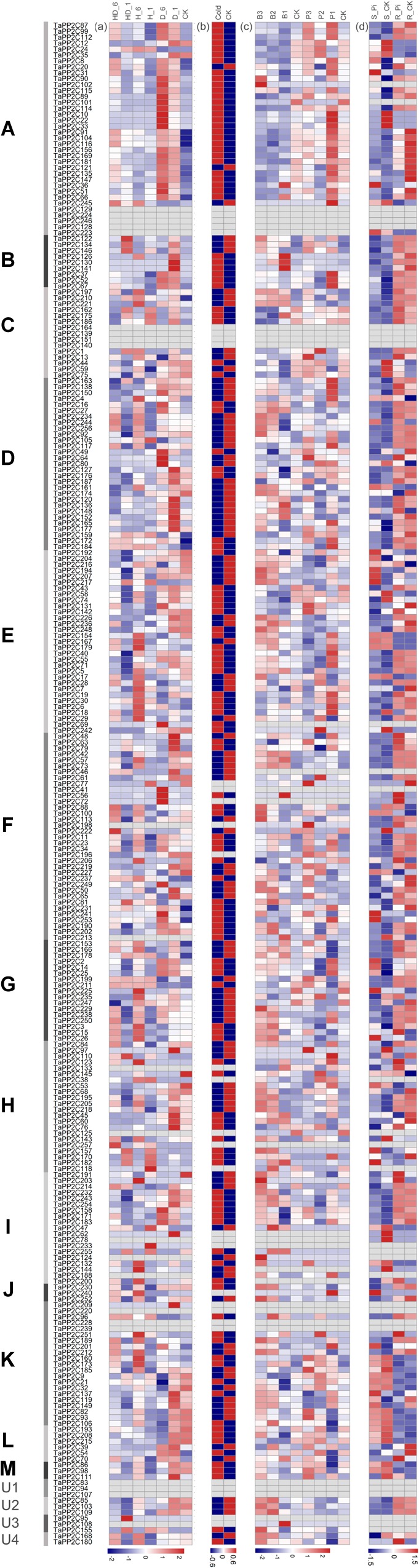
Expression patterns of *TaPP2C* genes under different stresses. Heatmap of *TaPP2C* RNA-seq data under different stresses was created by R program. Expression profiles were clustered according to the groups of all TaPP2C homoeologs. **(a)** Expression profiles under heat and drought stresses. HD_6, HD_1: heat and drought treatments of 6, 1 h; H_6, H_1: heat treatment of 6, 1 h; D_6, D_1: heat treatment of 6, 1 h. CK stood for control check. **(b)** Expression profiles under cold stress. **(c)** Expression profiles under stripe rust (*Puccinia striiformis* f. sp. *tritici*; *Pst*) and powdery mildew (*Blumeria graminis* f. sp. *tritici*; *Bgt*) stresses. B3, B2, B1: infected with powdery mildew pathogen at 1, 2 and 3 days post infection (dpi), respectively; P3, P2, P1: infected with stripe rust pathogen at 1, 2, and 3 dpi, respectively. **(d)** Expression profiles under Pi starvation stress. S_Pi, S_CK: stems of 10 days Pi starvation and control check; R_Pi, R_CK: roots of 10 days Pi starvation and control check. The scale legend lies down the corresponding heatmap.

To further investigate the potential responses of *TaPP2C* genes to different stresses, RNA-seq data of four abiotic (heat, drought, cold, and phosphate starvation) and two biotic (stripe rust and powdery mildew) treatments were acquired. These expression profiles were clustered according to the groups of *TaPP2C* homoeologs to identify the potential biological roles of each group (Figure 5). Several homoeologs with missing expression data under all treatments were observed; these are displayed as blank cells in Figure 5. Comparison of data shown in Figure 5 with those shown in Figure 4 revealed that homoeologs displayed as blank cells either maintained relatively low expression levels in all 15 tissues or preferentially expressed in particular tissues; most RNA-seq data were obtained from leaves of wheat seedlings, which may explain the presence of blank cells (represented by *TaPP2C78, 155*, and *223*). However, blank cells could also be the result of expression induced under a specific condition.

All *TaPP2C* genes showed varied expression patterns after cold treatment except those representing blank cells (Figure 5). Most of the genes in group A were significantly up-regulated after drought and cold treatments but not in response to heat treatment, with the exception of *TaPP2C24* (homoeolog of *TaPP2C-a2*) and *TaPP2C245* (homoeolog of *TaPP2C-a11*), which distinctly responded to heat stress but to not drought stress. Interestingly, the expression pattern of *TaPP2C24* was contrary to that of *TaPP2C13* and *TaPP2C38* (the other two homoeologs of *TaPP2C-a2*), probably because of differences in the upstream regulatory regions of these genes. Additionally, most genes in of group A were up- and down-regulated in response to powdery mildew infection and phosphate (Pi) starvation, respectively, indicating their roles in fungal pathogen defense and Pi metabolism. Thus, group A PP2Cs are involved in several plant processes, beyond the ABA signaling pathway. All genes in group B responded to drought stress and pathogen infection but showed no remarkable changes under Pi-stress. Several genes in group C were up-regulated under heat and drought stresses, such as all three homoeologs of *TaPP2C-c2*. The expression patterns of genes in groups E, H, and I under these stress conditions were similar to those of genes in group D, and 50% of the genes in these groups were significantly up-regulated by heat treatment. Genes in groups F and K exhibited diverse expression patterns under heat and drought treatments, but all of these genes were up-regulated upon fungal disease infection.

### Homoeologous Expression Patterns of *TaPP2C* Genes Under Stress Conditions

**FIGURE 6:**
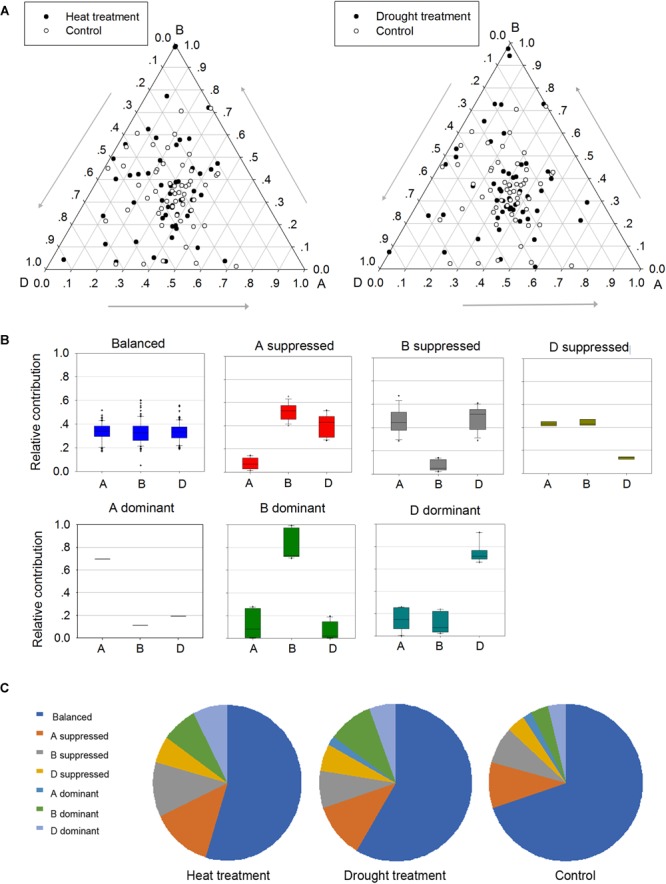
Homoeologous expression patterns of *TaPP2C* genes under stress conditions. **(A)** Ternary plot shows relative expression abundance of *TaPP2C* genes under heat and drought stresses compared with non-stress condition. Each circle represents a gene triad indicating the relative contribution of each homoeolog to the overall triad expression. **(B)** Seven categories of homoeologous expression patterns are illustrated as box plots. **(C)** Pie chart represents abundance of homoeologs from each subgenome on the basis of the seven categories.

To better understand the roles of homoeologs in *TaPP2C* gene family under different stresses, a comprehensive analysis of expression patterns was conducted (Figure 6). Firstly, genes in possession of triad homoeologs were selected from all *TaPP2C* genes. Then heat or drought stress treatments with the lowest number of blank cells were selected. Finally, 159 homoeologs (belonging to 53 *TaPP2C* genes) were put into analysis. Relative expression abundance of homoeologs within triads before and after heat and drought treatments is shown in Figure 6A. A simple impractical assumption was that each homoeolog contributed equally to the total amount of mRNA of a gene (Leach et al., 2014). In fact, nearly one third of *TaPP2C* homoeologs displayed expression bias within triads under normal condition, and this bias was more evident under heat and drought stresses, suggesting unbalanced functions of homoeologs in stress responses. According to their expression abundance, the unbalanced homoeologous expression patterns were divided into six categories: homoeolog-dominant or homoeolog-suppressed (Figure 6B). Overall, homoeologs from the D subgenome within triads had slightly higher abundance than those from B and A subgenomes, with more D-homoeolog dominance and less D-homoeolog suppression. Moreover, D-homoeolog dominance within triads increased obviously after abiotic stress treatments, especially after heat treatment. Additionally, B-homoeolog dominance within triads was notably enhanced under stresses, particularly drought stress, while A-homoeolog dominance was maintained at the lowest level consistently throughout all the treatments. These results showed significant variation in homoeologous expressions among *TaPP2C* genes, and stresses either increased or decreased the expression abundance of homoeologs of the same gene.

### Expression Analysis of Group A *TaPP2C* Genes by qRT-PCR

**FIGURE 7:**
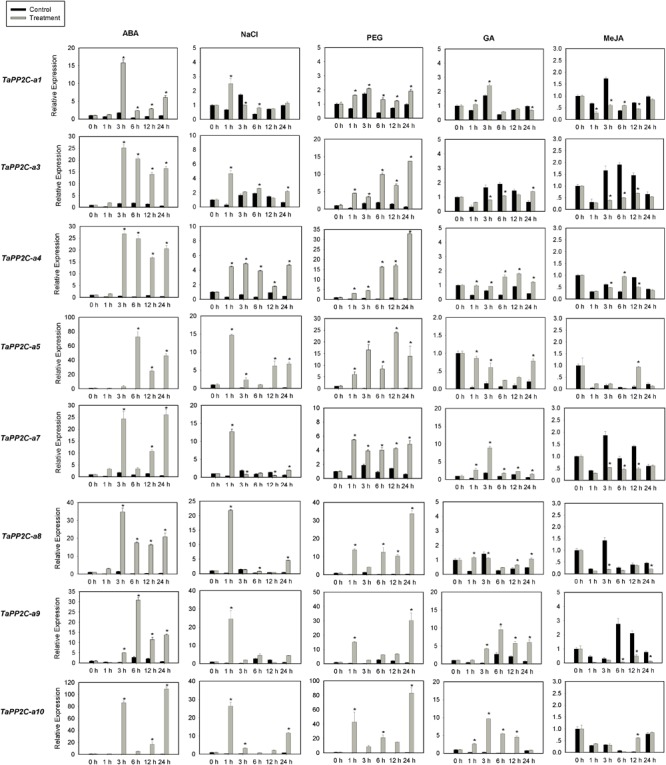
Quantitative real-time PCR analysis of group A *TaPP2C* genes in response to ABA, NaCl, PEG, GA and MeJA treatments. Error bars represent the S.D. of three independent replicates. An asterisk indicates significant difference between the stress conditions and the control condition (^∗^*P* < 0.05, Tukey test).

Several members of group A PP2Cs have been shown to function as negative regulators of ABA signaling pathway in *Arabidopsis*. To evaluate functions of *TaPP2C* genes in group A, the expression of these genes was examined by qRT-PCR under ABA, salt and drought stress treatments (Figure 7). Eight genes from group A were randomly selected for this analysis, and specific primers matching one or two homoeologs of the same gene were designed based on the expression patterns of *TaPP2C* homoeologs described above. Primers used for qRT-PCR were listed in Supplementary Table S4. Under ABA stress, the expression of all eight genes was up-regulated by more than ten-fold, which was consistent with the findings in rice and *Arabidopsis*. Seven genes were up-regulated under both drought and salt treatments, whereas *TaPP2C-a1* showed only subtle changes under both stresses. Expression levels of *TaPP2C-a8, -a9*, and *-a10* genes were dramatically increased after ABA, salt, and drought treatments; thus, these genes are excellent candidates for functional characterization in future studies.

Since group A *PP2C* genes mediated the crosstalk between ABA and other phytohormones in *Arabidopsis* (Manohar et al., 2017), we investigated the responses of group A *TaPP2C* genes to GA and MeJA (Figure 7). The results showed that *TaPP2C-a5*, -*a7*, -*a9*, and -*a10* genes were up-regulated to different extents after GA treatment, and *TaPP2C-a3*, -*a7*, -*a8*, and -*a9* were down-regulated after MeJA treatment.

### Protein Interaction Between Group A TaPP2Cs and TaSnRK2s

**FIGURE 8:**
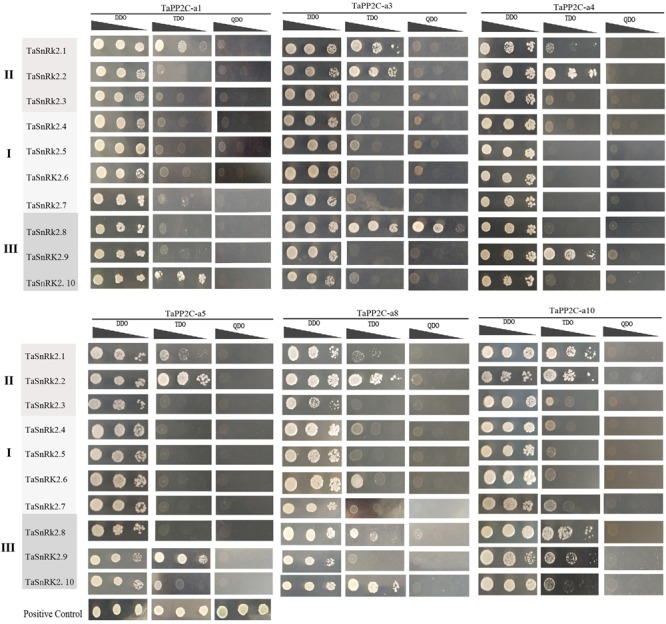
Yeast two-hybrid analysis of group A TaPP2Cs and TaSnRK2s. Positive transformants were cultured on selective medium DDO (SD/-Leu/-Trp), TDO (SD/-Trp-Leu-Ade) and QDO (SD/-Trp-Leu-His-Ade) separately. Interaction between SV40-T and p53 was used as a positive control. Yeast strains were assessed at different dilution rates (1, 1/10, and 1/100).

To systematically assess the interactions between group A TaPP2Cs and TaSnRK2s, we performed yeast two-hybrid assays. Ten *TaSnRK2* members have been isolated previously (Zhang et al., 2016). However, in this study, we found that the originally identified *TaSnRK2.7* gene was a homoeolog of *TaSnRK2.6* gene, while another gene identified as *TaSnRK2* gene was renamed *TaSnRK2.7*. The re-identified *TaSnRK2*s were presented in Supplementary Table S5. According to the result of qRT-PCR analysis of group A *TaPP2C* genes, six corresponding *TaPP2C* homoeologs were successfully cloned. Primers used were listed in Supplementary Table S4. The results showed that all six TaPP2Cs interacted with one or two members of subclass III TaSnRK2s (Figure 8). However, weak interaction was detected between TaPP2Cs and TaSnRK2.1 and TaSnRK2.2, which belonged to subclasses II SnRK2s.

### Ectopic Expression of *TaPP2C135* in *Arabidopsis* Enhances Its Tolerance to ABA

**FIGURE 9:**
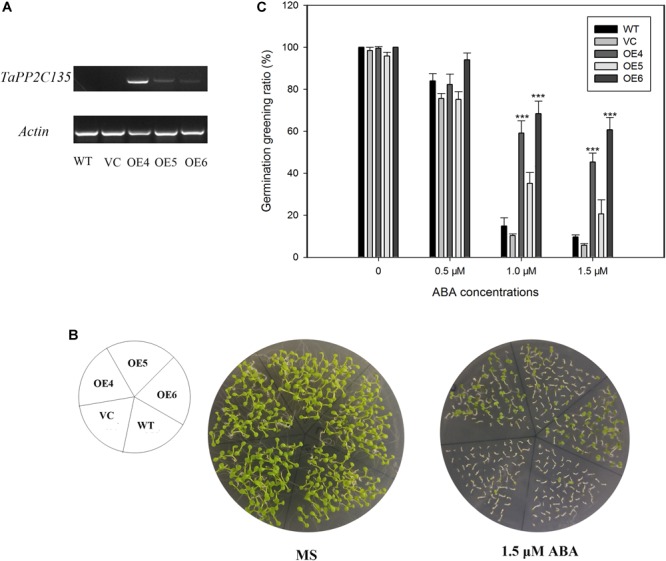
Ectopic expression of *TaPP2C135* in *Arabidopsis*. **(A)** Expression levels of *TaPP2C135* in the transgenic lines and the wild type. **(B)** Seed germination of transgenic lines and the wild type on MS medium with or without ABA. **(C)** Statistical analysis of the germination greening ratio in **(B)**. Error bars represent the S.D. of three independent replicates. The asterisks indicate significant differences compared with the wild type (^∗∗∗^*P* < 0.001, Tukey test).

*TaPP2C-a9* was greatly induced by ABA treatment (Figure 7). Therefore, transgenic *Arabidopsis* plants expressing *TaPP2C135* (B homoeolog of *TaPP2C-a9*) were generated, and lines OE4, OE5 and OE6 were randomly selected for further analysis (Figure 9). Transgenic *Arabidopsis* of pSN1301 vacant vector (VC) was used as control. Expression levels of *TaPP2C135* in the transgenic lines and the wild type were verified by RT-PCR (Figure 9A). The seed germination greening ratios of transgenic lines and the wild type on MS medium with or without ABA treatment were calculated (Figure 9B,C). While on MS medium, no difference was found among *TaPP2C135* transgenic lines, VC transgenic lines and the wild type. With increasing ABA concentrations in the medium, the seed germination greening ratio significantly decreased. However, the seed germination greening ratios of *TaPP2C135* transgenic lines were higher than those of the wild type and VC controls, especially at higher ABA concentration, indicating that *TaPP2C135* transgenic lines were more tolerant to ABA than the wild type and VC controls.

## Discussion

PP2Cs play important roles in various stress signaling pathways. Plant PP2Cs function in response to stresses such as drought, salt, alkali, fungal pathogens (Bhaskara et al., 2012; Chen et al., 2017; Akimoto-Tomiyama et al., 2018) as well as in plant development (Yu et al., 2003; Song et al., 2006; Fuchs et al., 2012; Ren et al., 2018). However, limited research has been conducted on PP2Cs in wheat, and the only report on *TaPP2C1*, belonging to group F, is based on its role in resistance to salt stress in transgenic tobacco (Hu W. et al., 2015). Recent advances in the genome sequencing and annotation of allohexaploid wheat (Brenchley et al., 2012; Clavijo et al., 2016; Appels et al., 2018) have facilitated the analysis of gene families in wheat at the genome-wide level. In the present study, we performed a comprehensive analysis of *TaPP2C* genes in hexaploid wheat including genome-wide identification, chromosomal locations, synteny relationships, gene structures, conserved domains, motifs and expression patterns under diverse stress conditions.

PP2Cs have been evolutionarily conserved from prokaryotes to higher eukaryotes with an increase in the diversity and total number of genes during evolution (Fuchs et al., 2012). A total of 257 homoeologs of 95 *TaPP2C* genes were identified. The uneven distribution of homoeologous genes across different homoeologous chromosomes was probably caused by evolutionary events such as gene duplication and chromosomal translocation (Clavijo et al., 2016). These TaPP2Cs were further divided into 13 groups by phylogenetic analysis with rice. Our results showed that the wheat genome harbors a higher number of *PP2C* genes than rice and *Arabidopsis*, especially in groups A, H, and K, suggesting a greater diversity in the biological functions of TaPP2Cs. Known chromosome translocation events and pericentromeric inversions were found to involve in the formation of the *TaPP2C* gene family. Additionally, synteny relationships of *TaPP2C194/207/217* and *TaPP2C195/205/218* indicated potential pericentromeric inversions between the arms of chromosome 6B, while this event has not yet identified (Devos et al., 1993). Understanding the evolutionary events involved in the formation of the *TaPP2C* gene family facilitated further analysis of homoeologous expression patterns. The Ka/Ks ratios of groups A-I and K indicated that although divergence took place after duplication, genes in these groups retained their structure and function under selection pressure.

Although the distribution patterns of exon–intron structures of most genes in the same groups were similar, there were several exceptions, which could be attributed to various reasons, such as individual intron loss, gain or sliding during the formation of the *TaPP2C* gene family (Rogozin et al., 2005). Unlike other members in the same group, *TaPP2C-a8, -k1, -k2, -k3* contained only one exon, suggesting intron loss during evolution. The absence of introns from genes would accelerate evolution by gene duplication (Lecharny et al., 2003; Cao et al., 2016), which could explain why group K contained more number of genes in wheat than rice and *Arabidopsis*. On the other hand, alternative splicing, which is common in post transcriptional processes of eukaryotic genes, could create additional mature mRNA transcripts (Koralewski and Krutovsky, 2011). While cloning *TaPP2C35* and *TaPP2C89*, three and two transcripts were identified from these two genes, respectively, which confirmed the alternative splicing of *TaPP2C* genes.

Eleven conserved motifs were found among TaPP2C amino acid sequences. Proteins in the same group exhibited similar motif distribution patterns. Nine motifs were conserved in TaPP2Cs, OsPP2Cs, and AtPP2Cs, while the other two motifs were specific to TaPP2Cs. The majority of TaPP2C proteins displayed a similar motif pattern, i.e., motif 4, followed by motifs 7, 3, and 1; this pattern was closely related to the catalytic core domain of PP2C proteins (Shi, 2009; Marchler-Bauer et al., 2016). The motif distribution pattern provided a reference for the functional analysis of genes in different groups.

The spatial and temporal expression analysis of *TaPP2C* genes revealed tissue- and stage-specific expression patterns. Further expression analysis showed that most *TaPP2C* homoeologs responded to heat, drought, cold, Pi starvation, stripe rust and powdery mildew treatments, indicating that PP2Cs in different groups participate in the same stress response. Common wheat is a vernalization-requiring plant that exhibits higher tolerance to low temperatures during the vegetative growth stage. Therefore, TaPP2Cs may contribute to cold tolerance in wheat. Group A PP2Cs function as ABA co-receptors in regulating seed germination, and responses to salt and drought stresses. Interestingly, our results revealed that most TaPP2Cs in group A not only responded to drought stress but also to fungal pathogens, which has not been reported previously. Two *TaPP2C* homoeologs in group A were unexpectedly up-regulated by heat treatment but not by drought treatment. A previous study demonstrated that almost all members of group D in soybean (*Glycine max*) and *Arabidopsis* contain SA responsive element, heat stress responsive element (HSE), and MYB binding site involved in drought-inducibility (MBS) in their promoters (Chen et al., 2017). This finding is consistent with our results that genes in group D responded to pathogen infection as well as heat and drought treatments. Similarly, expression of genes in groups F and K were consistent with previous studies, thus showing the role of these genes in plant immunity and pathogen resistance (Lee et al., 2008; Akimoto-Tomiyama et al., 2018). Therefore, both conserved and divergent expression patterns of *PP2C* genes exist not only in wheat, but also between wheat and *Arabidopsis*.

Analysis of homoeologous expression patterns exhibited differences under different environments, thus providing important information for the functional analysis of *TaPP2C* genes. Moreover, relative expression abundance of homoeolog from the D subgenome was slightly higher than that of the other two homoeologs within a triad, which is in agreement with the finding of Ramírez-González et al. (2018). While most *TaPP2C* homoeologs within triads acted redundantly to display dominant effects of genes, in some cases, a single homoeolog showed a predominant effect under normal conditions or stress treatments. For instance, the *TaPP2C-d9* and *TaPP2C-k6* triads in balanced category under normal condition shifted to B-homoeolog dominant and D-homoeolog dominant category after heat and drought treatment, respectively (Figure 6B). Thus, homoeologous expression pattern is of great importance to target and manipulate individual or multiple homoeologs and quantitatively modulate agronomic traits for crop improvement.

In plants, ABA plays a vital role in regulating developmental process, and responses to abiotic stresses such as salt and drought (Fujii and Zhu, 2009; Yoshida et al., 2014; Hu W. et al., 2015; Zhang et al., 2017). Expression analysis by qRT-PCR of *TaPP2C* genes in group A revealed that they were all up-regulated after ABA treatment. Some of the genes in group A also responded to other phytohormones such as MeJA and GA. Analysis of upstream regulatory sequences revealed the presence of ABA-, GA- or MeJA-responsive elements in the promoters of these genes (Supplementary Figure S2), suggesting that these genes function in other phytohormone signaling pathways in addition to that of ABA. SA suppressed ABA-enhanced degradation of group A TaPP2Cs (Manohar et al., 2017). Brassinosteroid (BR) signaling was inhibited by ABA signaling with the participation of ABI1 and ABI2 (Wang H. et al., 2018). However, all group A *TaPP2C* genes tested above, barely responded to BR treatment (Supplementary Figure S3), probably because of homoeolog-dominant responses of *TaPP2C* genes to BR stress.

In *Arabidopsis*, group A PP2Cs negatively regulate the ABA signaling pathway by binding to subclass III SnRK2s (Umezawa et al., 2009, 2010). However, limited data on this interaction are available in monocot plants (Wang et al., 2015; Zhang et al., 2016). In this study, results of yeast two-hybrid assay showed that group A TaPP2Cs interacted not only with subclass III TaSnRK2s, but also with TaSnRK2.1 and 2.2 in subclass II TaSnRK2s. Expression analysis of subclass II *TaSnRK2*s after ABA treatment revealed that these genes responded to ABA stress, although less remarkably than subclass III *TaSnRK2*s (Zhang et al., 2016), suggesting potential roles of group A TaPP2Cs together with subclass II TaSnRK2s in ABA signaling pathway in wheat. This result differs from the finding in *Arabidopsis*, but is consistent with a previous study in *B. distachyon* that a group A BdPP2C interacted with BdSnRK2.1 (Wang et al., 2015); thus implying the differences in networks of SnRK2s and PP2Cs between monocots and dicots. Further investigation of the relationship between group A TaPP2C phosphatases and SnRK2 kinases in ABA signaling pathway is needed.

*TaPP2C135* was transformed into *Arabidopsis* for further functional analysis. Germination assay of *TaPP2C135* transgenic and control lines revealed that ectopic expression of *TaPP2C135* in *Arabidopsis* enhanced its tolerance to ABA. In *Arabidopsis*, the group A PP2Cs are classified into two subfamilies: ABI1 and ABA HYPERSENSITIVE GERMINATION1 (AHG1) based on their sequence similarity (Nishimura et al., 2018). While *abi1* and *abi2* mutants showed increased ABA tolerance, *ahg1* and *ahg3* mutants displayed a strong ABA hypersensitive phenotype in germination (Leung et al., 1994, 1997; Yoshida T. et al., 2006; Nishimura et al., 2007; Wang K. et al., 2018). Sequence alignment of TaPP2C135 with the AtPP2Cs in group A showed that TaPP2C135 was closely related to the AHG1 subfamily (Supplementary Figure S4). This is consistent with the germination phenotype of *TaPP2C135* transgenic plants. Our results indicate that TaPP2C135 is involved in the ABA response.

Overall, in this study, genome-wide identification and basic functional analysis of *TaPP2C* genes family in wheat were conducted. These results provided novel insights into *TaPP2C* homoeologs, particularly genes in group A, and provided with useful clues for further functional characterization. In our subsequent research, the functions of *TaPP2C* genes in group A, especially *TaPP2C-a7*, -*a9*, and -*a10*, will be further validated by gene overexpressing or gene silencing in wheat.

## Author Contributions

GH and GY conceived the study. XY and JH collected the data. XY and EW conducted the experiment. JX and RH participated in the data analysis. XY wrote the draft manuscript. GY and GH revised the manuscript.

## Conflict of Interest Statement

The authors declare that the research was conducted in the absence of any commercial or financial relationships that could be construed as a potential conflict of interest.

## Footnotes

1 https://www.arabidopsis.org

2 http://rice.plantbiology.msu.edu/

3 http://www.ncbi.nlm.nih.gov/

4 http://plants.ensembl.org/index.html

5 https://www.wheatgenome.org/

6 http://hmmer.org/download.html

7 http://pfam.xfam.org/

8 http://smart.embl-heidelberg.de/

9 https://www.ncbi.nlm.nih.gov/cdd

10 https://sourceforge.net/projects/kakscalculator2/

11 http://gsds.cbi.pku.edu.cn/

12 http://www.wheat-expression.com/

13 https://www.r-project.org/

14 http://www.sigmaplot.co.uk/index.php

15 http://bioinformatics.psb.ugent.be/webtools/plantcare/html/

## Supplementary Material

FIGURE S1 Phylogenetic analysis of TaPP2C and OsPP2C proteins. A total of 257 TaPP2Cs and 80 OsPP2Cs were used to construct the phylogenetic tree using the NJ method with ClustalX 2.1 and MEGA 6.0 software. The PP2C proteins were grouped into 13 distinct clades (A-M), which were indicated with different colors except for the ungrouped PP2C proteins.

FIGURE S2 Quantitative RT-PCR analysis of group A *TaPP2C* genes under BR treatment. Error bars represent the standard error of the mean of three independent replicates.

FIGURE S3 Promoter sequence analysis of *TaPP2C* genes in group A.

FIGURE S4 Sequence alignment of TaPP2C135 with the AtPP2Cs in group A.

TABLE S1 The *PP2C* gene family of wheat.

TABLE S2 The classification of TaPP2C proteins.

TABLE S3 The Ka/Ks ratios of *TaPP2C* gene pairs.

TABLE S4 All the primers used in this study.

TABLE S5 The re-identified *SnRK2* family in wheat.
