# Supplementary figures and images for: Genome-Wide Identification and Homoeologous Expression Analysis of PP2C Genes in Wheat (Triticum aestivum L.)
Source: Front Genet. 2019 Jun 12;10:561. doi: 10.3389/fgene.2019.00561 (PMC6582248; doi:10.3389/fgene.2019.00561)

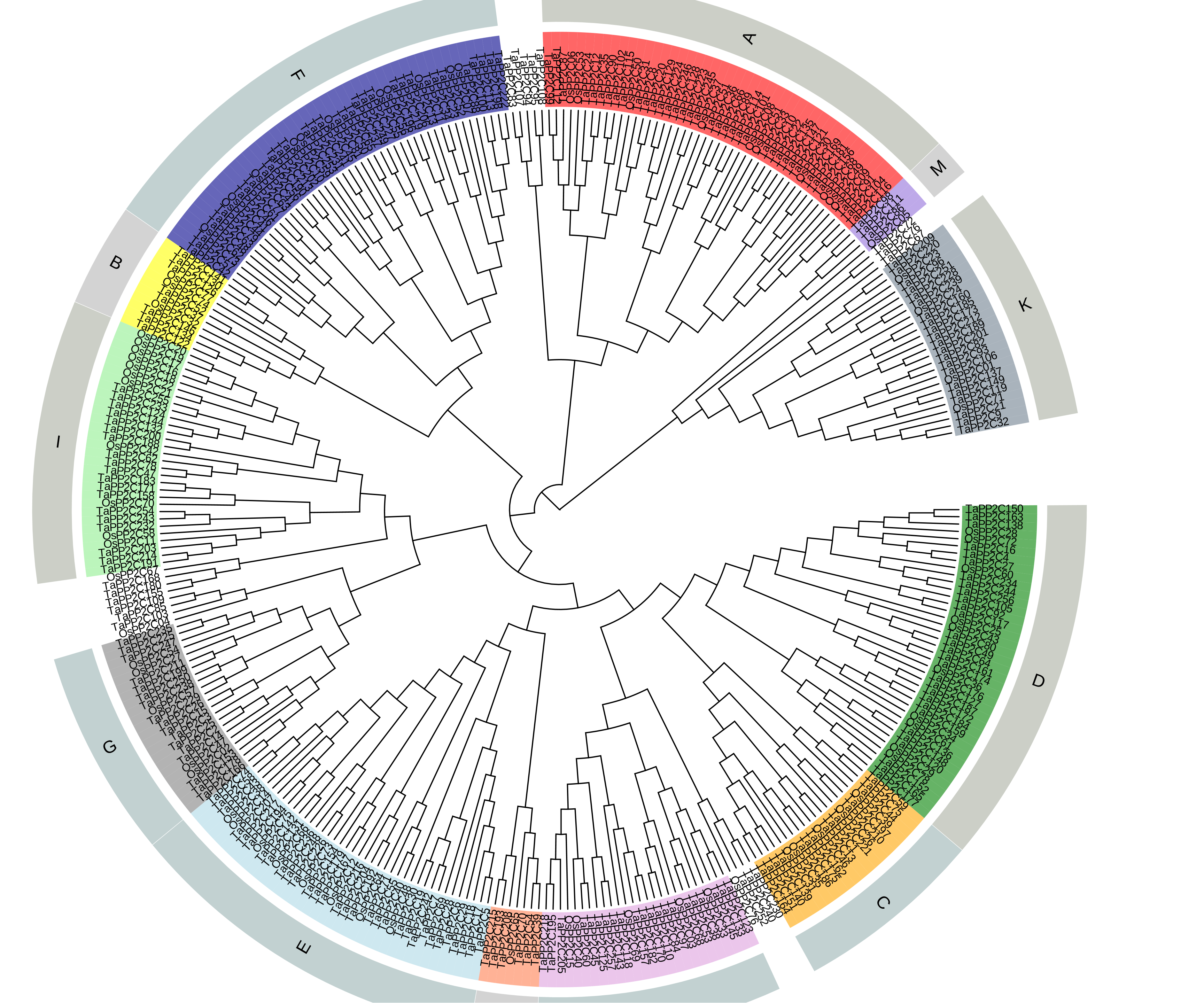

Supplement: FIGURE S1 — Phylogenetic analysis of TaPP2C and OsPP2C proteins. A total of 257 TaPP2Cs and 80 OsPP2Cs were used to construct the phylogenetic tree using the NJ method with ClustalX 2.1 and MEGA 6.0 software. The PP2C proteins were grouped into 13 distinct clades (A-M), which were indicated with different colors except for the ungrouped PP2C proteins. [file Image_1.TIFF]

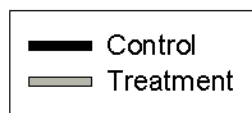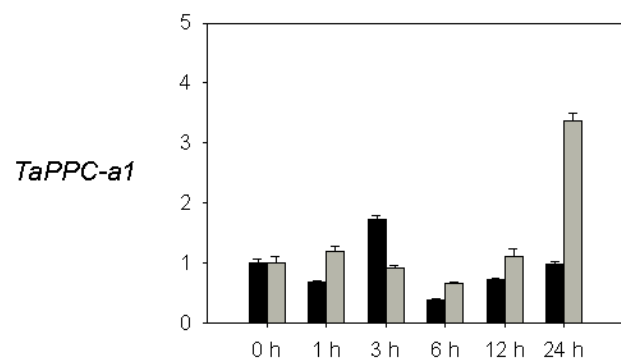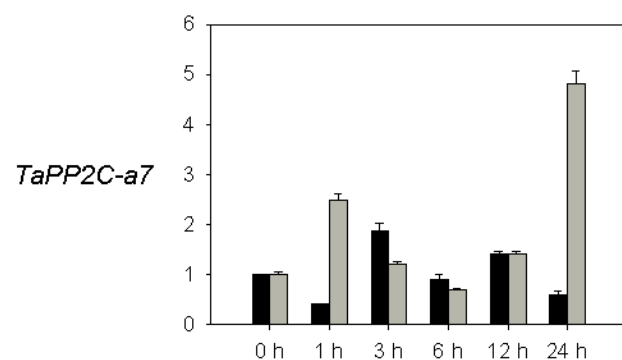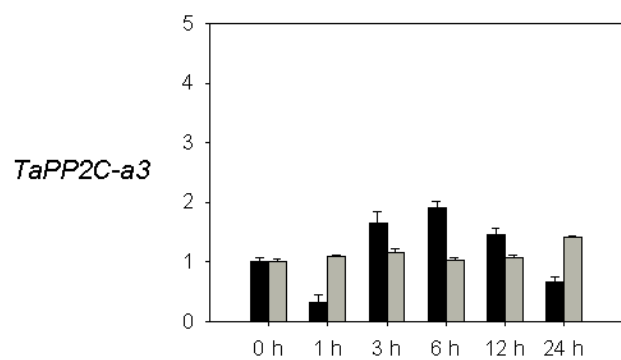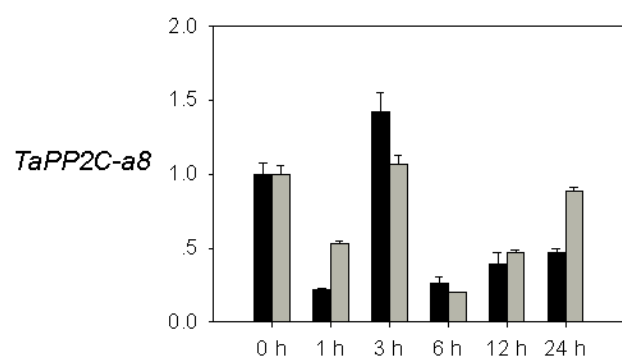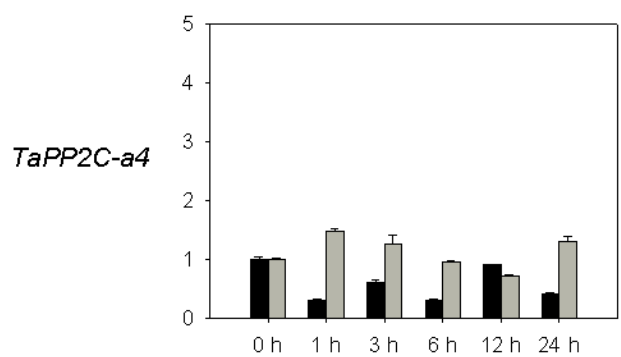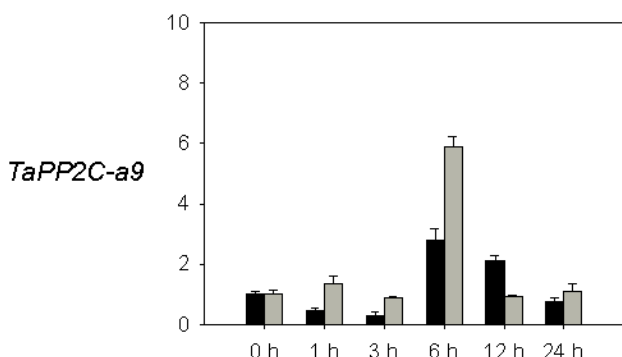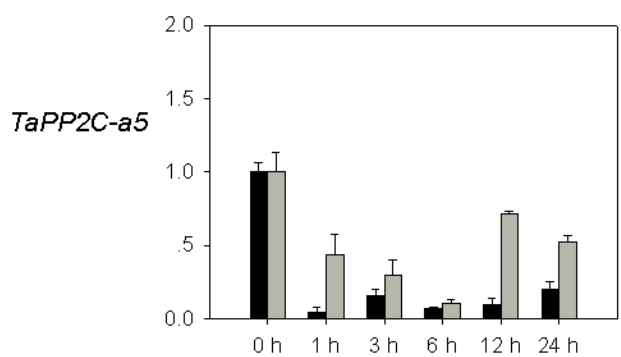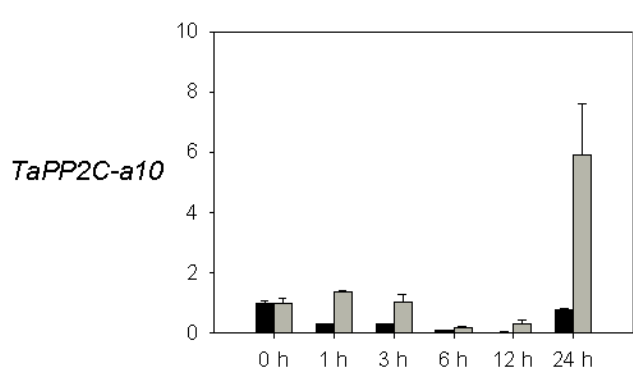

Supplement: FIGURE S3 — Promoter sequence analysis of TaPP2C genes in group A. [file Image_3.pdf]

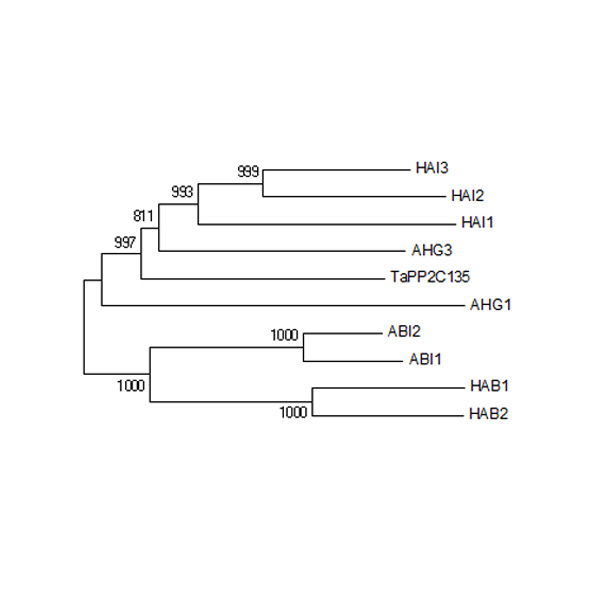

Supplement: FIGURE S4 — Sequence alignment of TaPP2C135 with the AtPP2Cs in group A. [file Image_4.jpeg]
