# Supplementary material for: Genome-Wide Identification and Homoeologous Expression Analysis of PP2C Genes in Wheat (Triticum aestivum L.)
Source: Front Genet. 2019 Jun 12;10:561. doi: 10.3389/fgene.2019.00561 (PMC6582248; doi:10.3389/fgene.2019.00561)
Supplement: FIGURE S2 — Quantitative RT-PCR analysis of group A TaPP2C genes under BR treatment. Error bars represent the standard error of the mean of three independent replicates. [file Image_2.pdf]

: ABA-responsive element

: GA-responsive element

: MeJA-responsive element

>*TaPP2C-a5* (*TaPP2C89*) promoter

GCATTATTGTTGTTTTCCTTCACCATAAGCTGATGCACAAGGAATGTAACTGACCACTGATGGACGGCTTCAATATAACACCTTTGCACTCTTTAAATGCCTTTTGTC
TTTTATAATGTCTATATACCACGGAGTCTGTTTCGGCGCGTATAGAAAGATAAATGTGTGCCGATGAATTCAAATAAGTTATAGTCATTATATAACTGATTGTGAATT
GATTTAATGAAGTTATAATGCCTTGTTGTTGTTGTGTAGTGTGTTTGTCCTTTGGGGCGTTAGCACGTCGACTTTCTGGATGTCTACTTCAACAAGGTTTGTTCGACTG
CTGTGAAGGAGGGGTGATGACGGCGATGCCTCTTCAGCTCACTTCAATGCTTACAGTCGTTGTTAGATGATATGGATATAGTTTTTGTTACCGTTGTGGTCTTTGTA
CTGCCATGATGTGATGAATAGATCAGAAGTCATCCCCACAAAAAAATGGTTTCTCGAATGTTGTATACCCGCTACTAATTTATTACTAATGTCATTATTACTTCCTCT
GTCCGGGTTTATAGATGGTAATAGTACTGAAATGTTTGATACCTATCACTAATTTATTACCGGTGCCATTCTTACTTCCTATGTTTGAGTTTATAAACCCATATTGTAT
TTTGCATAAAATTTAACCATGGGTGGGCTAGCAAAATATATTTATTTATTTAAAAATATCATGTATTTGTATTTGAACTTTTTTATATTGGTATTATTTTACTACATACTC
CCTCTGTCTCAAAATATAAGATATTTTTGGTCTAAATCTAGAACAAAAAACGTCTTTATTTTAAGATAGGAGAGTATAATTTATGTTTTTATTAACCAAGTTTATGATA
AAAAAAATGACTGAAATACAAGGGGACTAACAGAAACGAAGAAAGGGAGTACCAATTTTGAAGATGATGCATTTGTGAATGCCTATCAAAACCTAACGAGTGCC
TCTTTGATTGATTTGTAAAGTAGAAAAAACACAGGAAGAGGGTGTCTGCCATATTCGTATATAGGATTGCTCCGATGTTTGATTGAGCACATGAAAACACATAGAA
ATCTTCATATAAGATTGAAGTGGATGAAAATTTATCTATGAAATCTAGATCAAGTGAATCTTGGATTACATTCCTACGAGTCCAACGTTGTGTGTAGGGGAAAAAAA
ATCTAAGGATCAAAATCTTCAAAAATACCGTCACGAATCATTTGAATCAAAGAGGTCTTGGGTGAGTAGTGACAGCTAGGGTTTTGGTTACCGCTTGAGAAATTCG
GTTACGGGGGCAGATTGATGCCCACGAGAAATCACCTTACCGAGCAGGATATCCCAAAAACAGTGAATTTAACTGAATTTTGACTGAATATTATCCAATTTTAAAA
TGTATTCAAAATTGCGTTCTCCACAGTAACCAAAAACCTCAATGCTTCATGAGATCTTATTTTCATCGGGGTCCAAAACCTTGGTGACAGCTTGTGTGCATCTACCA
GTTCTTGTCTACCAGTCAAAACTGCACAACATGCCCCACAGGCGTCATTCCTCTCCCATCCACGTAACCCCTCCCACTTTCAATTCAGGTCCCACGCGTTACCTAAA
AGCGCCAAACGTTAGATCGTTCCCTGGACCCTCCCCACAAGCACCGCAAGCGACTAGAAAGCCTGGACCCGCCGCCAGAAGCCGACGCCCCTCTCCACGGTCCA
TATGAGCCGCGCGTACCACGCCCGTCCGCGAGCCCCGAGACTCCGAGCCGCTCATCGCGCTCGCCACGCGCCTGCCGCCGGCTCATCGCCGCCGTCCACGTCCA
GGCCCGCGAGATCCACGTCCGGATACGGCTCCCGCCCTCGCTGTCACGTATCGGGGCCATCTACGGGAAATCGGACGGCCCTGGAGCGCTGTCTAACCTTTTCC
CTCCCGTGGCCGCGGGTATTAGAGGCGCCTCGCCGTCCCACGCCCACAGTTCTTTTTGTTCTCTCCGGCGAAGGAAATGAGCAGCGAGACGAGCAAGCGAGA

>*TaPP2C-a7* (*TaPP2C116*) promoter

CATCCTACTGATGTGGTCATGGACGACCAATGCAACCACCATTATGTCCTCATTGTCCGATGAACAAATGAAGTTGTGAAAGAAGTACTCATCTCCGCTATCCATG
GTACCTTGCAAGCAAAGCGTCGAACACCTTGCGGGCGTGGTGGAGACGTGGCCGGAAAGGGCATGTCGAGACCGTTGGTTCGCAGCAAGGTGGTCGACCATGG
GCGTGGCGGAGGGCGGAGCGATGACTGCGACGGAAAAAAGGGGCTGCTCCACCGACGGGCAAAGCAAGGTGGTGACGGGCGTGCCGGCGCCGATGGCGAGG
GGCCGAGGGCGGAGCAACGAAGGGAAGGAAGAAATGGGGTTGTGTTTCCGACGGACGTGTCAGAAGAGGACAAGGGCGAGCGTCCCATCCGTCCGCGTGATG
TCCGTTTGGCCGCAAACGCGACCCAAAATTGGGCCAGGAATGAATCGAAAACGGACCGAAAATTAACATTTGTTCTTTTACTCTTGCGCGTTGGGCCGAGGTTTGC
GTTGGTTTACTCCAAACAGACGCGGGCGAACGATCTGGGCCGTGCGTACGAGCTGCCCTCACTCTACGTGGTAGTTCAGTGGTACTGAGACCGCACAGTAGCCG
CGACAACTGCCACACAACACGTAGTGCGTCCCTCCACTGCAGGAAGCACTGTTCAGCTGCATGCCGCGCCTGTCAGCCCCGCCGAAGTGCGCGCCAATGGTTTC
CCACGCCGAGCCGACGATCGGTGGTCACGCCACACAACGGCGCCAGTTCCGCCACGCAGATCCATCTCTCTCAGCCACGCACGCGCGCCTTTCCCCGAGGTTCA
CCTCCCCTTCGCCCCCACCGCAACGTGGCCACGCCAGCCCCCAACTACCAGCCGCCTTCCCCCTCCCCCACCCCCACGCAACGGAGCACACCAAAAGGCACCCC
CGGCCTCCACGCCACGCGTCCCCTCCTCCCACGACCGACCAACCTCCCACCGATCGCGACCGCCCCTTACCACGTGTCCCTCCCCCTCCACCCACCCGCCTCCCA
CGTCCACACGCCCCTACCCNNNNNNNNNNNNNNNNNNNNNNNNNNNNNNNNNNNNNNNNNNNNNNNNNNNNNNNNNNNNNNNNNNNNNNNN
NNNNNNNNNNNNNNNNNNNNNNNNNNNNNNNNNNNNNNNNNNNNNNNNNNNNNNNNNNNNNNNNNNNNNNNNNNNNNNNNNNNNNNNN
NNNNNNNNNNNNNNNNNNNNNNNNNNNNNNNNNNNNNNNNNNNNNNNNNNNNNNNNNNNNNNNNNNNNNNNNNNNNNNNNNNNNNNNN
NNNNNNNNNNNNNNNNNNNNNNNNNNNNNNNNNNNNNNNNNNNNNNNNGCCCCTACCCCCATCCACTGCCATGCGGGTCACCGAGCACCCATGGC
CCCGCCGGCCATCGACCCGTCGCTGCATGGACCGGTCAGCACGGCCGGAGTAGAATAACCCCGTGATATTTCCGGGCGGCACAGGAGGCACAGCCCATGTGTC
GCGGGCCCCGCGGGCATCAATAAAGAAGCTGCCGCGGCGGGCTGTCTCGCGGCGAGCCGCCACGTGTGTGTCTGGCGATAACACCGGCGTCGCCGCCGTTAGC
ATCAGCCTCCCGGTGGGGCCGAGATCGCGAGCACGTGTCGTCATCTGCCCGCCGGGCGCGCCCACGCGTCCAGATCAGGTGGAGACGGACGGCGAGCACGTCA
CGGACTCACGGCGACGGTAGCTTTCGGGCGCTCCCGCCATGACGGCGACGGGCACCCTCCCTCCCCTTCTTCTACCGCCCCACCCCGCGTGTGTGAGGTTCGTTT

CGGCTCGCGCCTCTGCTGCTGCTTAGCTTCTACTGCTGCCATTCGAGCTGGGAACGCGCGCGAAGGTGCTGTAGTGTGTCGAGGTGTTTCGTGTAGGCCTCAGGG
TTTCCGTCGCCGACGTGCGGCGGAGGGCAGTGGGAATCTAGGGCGGCGTCGGGGTCGAGCGAGCGATTACTTGCCA

>*TaPP2C-a8* (*TaPP2C169*) promoter

AAACAAAACCACTTTGCAACAAATGCAAGAAGCAACTAGCAAGGATGTGGAGAGGGGCATTCATAATGCTCCAAGCTCGTAGGAAATTGTTTGTCGAGCTGCAAT
GATGTTGGAATCGGACACTTTGTGACACCTGGTGACATATTGTGCCATTATGCACAATATAATTATCAAAGACGAGGGTGAAGGGGCAACTCGAACCCATGATTTG
AAGAAGCCCGGAGAAGAAGTCCACGCCCCAGATCAAGACGCAGATCATATTATGAATTTTCTGTAGATATGTCAGAATTTTCAAGATGAACAGGTGTGCGTGCAG
CTACTCCATAATCTTGTGGAGCATGTGGACCCACAATAAAAATCAAGGATGAAACCAAGAAGTTTAATGTTTGAATTGTTCACTTCATATAAATTTTATGTTTGAACT
ACTCAATATTTTGTGATATTCATGTGTATGCTGGACGGTTATATACATGGATTTAAAGATTTAGATATGAGGAGCATGGTTGTCCATAAGGACTTTTGAGGGGCCGT
TCAATGCTGTCCGCGGACGTTTGGGATGTCAGATTTCCTAAATCCGATTGGATGCTCTGAATGTCCGTTTGTTTATTAACATGGATTGTTGGGTATTTGCATCTGTGA
CTGGTTGATCTCGTTTGAGGGTATACATGCTCTAGACCACGTTTTGCATGGGGTTGTTCCCCTACATTCTTGCATCGTTCTCGTAGTGTAAATTGCATTAGTACATCC
GAACTGAGCGTTCCTGACGGACGACCGAGGGATGTTTTAAGTTTCATGCGGCGTGGTTTTTTCGTGCGAGTCAGCTAACCAAACAGTTTAAAACTTAGCCCCGAGA
GTCTGATTGCGCTTGATGTAGACAGCCCATTTTCGAGCTTTGAACTCGCCATTTCTACTGGCCCTGGACGCCACCTGTAGCTCGCCGCTTGCATATCCCCGCCGCTG
GCCCTCAACCCCACGGCACCCAATACAACCTGAATCACCCCGAAACAGCCGCAGTTTCCCCGGTTTCAGTCCCAAGGCGGGGCCCCGACGGTGACGCCCACTGT
TCCGCGGCGCCCCCACCTGGCCACCTCCCGCCGCATACGTGCCCCTCCTGCCTCCCCTGACGTGTCGACGACGACCAGAGAGAGCGGTAAAAAAATCTCGCCCC
TCGCCGGGGCGGGCCCCGCGCGGTTCCAGCCCTCGGGTGGCACGTAACATTGCGCGCCAACAAAGAGCACGACGTGTCACGTCTCCCCCGGCTCCGGTCGCCG
GAAAATTTAAAAACTGCGTCGTTGCGAGGTCAAACGGGACACGAGTCGCCCCCTCTGCGCTCTGGGGGGCCACGGCGCGTCTACGAGGTCGTCCCGGGCCCGA
GAGCCAATGGGGTGGCGGCACGGCGCGCGCAACCACCACCCCCAACCCCCGCTTTTTCATTTTCTTTTCTTTTCTTTTCGAATCCGCATCCGCATCATCAGATCCG
ACGGAACCTTTGCCCTGCCCCCCGAGTTGGACCGGCAACGGAGAATTTCGGGGAATCCCTCGCTCCGCCTAAACGGGACACGTCGCAGTGTTTTACACTCCCGG
GGCCACGATCGCGTGCCCGGTCGCCATCGCGCACCTGTGCCCGCATTATTCTATCCGGACGTCTGCTCGTTCGGCGCGATTGGGTTGGGGGGTTAGATTGGACTT
TCAACAAAAACACACTAGCTTAGATTTGATTATAGACTAGATTAGTTGATTATCGAGGGTGTTATCGTGGCCGCCTGTCACGTGTCCCGGTTGGGGAGAGGGAGG
GTGAAGGGTGAGCCGTTGATGGGGCGGGGCACGTGTCGTGGTGCGCGCCGCGTGCCATGCGCAACTCGTGGACGGCCCGGATGTTGCGTGGGGCGGTGGATGC
CACCATGTGTCGCCTCCGCCTGAGAGATGGCGACGCGCGCCATGGATGTGCCACGCCACCTGCGACTGCGCTTTATAAGCGGGCCGCCCACCAGAGACTCCTCT

>*TaPP2C-a9* (*TaPP2C121*) promoter

ACCAAACGACATGCAGAACATGGTTCAGAGCATGTATACACTTGAACAGACTCCATTGAAAATGTACTCAAACACGCCCTCGATGATCTTATCAAGTCAGTGCAA
GCATTTGTGTGGGAAAACAGGGGAAGGTTTCGCAGTTTTCGGGGAGAGATTTTTGAAGAGCCTTTTCTAGGGCTTTCCCACAGTTTTCTTACCATAATGCGCAAGCT
TTACGGTGCGGCCAATCACACGCAAGGAAAAATATCGCGCTGGAGAACATGGTCCAGATGGCCGGCCGGCTGTTATTCCGAATGCCCGTCGCACGCCACCGAAC
GAGCCCTGGCGCGATACGTGCCAGTGTGCCTGGCCACAAACTCCAAACAAAGTGCCGGAATCATGGAGCCCAGGACGTGTCCCGTGTGTGCCGTGTCTGCATGC
ATTGCCGCGGGCGAAAAGATCTACTAGCGCACACGGCACACCTGTCTGGCTCTCGGAGTTGCGGTCGTACTTGCCTGCCACCGGTATGGCGGTGACCTGGATGG
CTGGCTGTTCAGTGCAGGACTCCGTTCATCCATGAGTAGAAGAAAGCTCTCCACTTTCTGCTATCTGTTTCTCGCGCCAGCAGATTCGTGTCCGCATGCGATATAAT
GCTGATCAATTAAGCTTCAAATGCAGGAGTTCGCGCAGCACCAACGGCCGTGAAATGACGCGCAGAAAACTTAGGCTGGGTGGGTCTGGCCAGCTAGCTGTGTG
ATTACTCTATGCACATGCCCATACCCGACGCGTGGTTTTTTTTNNNNNNNNNNNNNNNNNNNNNNNNNNNNNNNNNNNNNNNNNNNNNNNNNNNNN
NNNNNNNNNNNNNNNNNNNNNNNNNNNNNNNNNNNNNNNNNNNNNNNNNNNNNNNNNNNNNNNNNNNNNNNNNNNNNNNNNNNNNNNN
NNNNNNNNNNNNNNNNNNNNNNNNNNNNNNNNNNNNNNNNNNNNNNNTTTTTTTGCGAATTACCCGACGCGTGGCTTGAGTATGTGATTAGGACGTC
GCTCATGCTCGAGACGGGCACGTCCTCCCTTTCTGTCGATGTGGATGGCTCCTCTCCTTTGTGCTGTGCACGTACGTGTCTGGCGTTCAGTTGAGTACATGGCATTG
TCACCACAAAATGGACGCCAGCAGCTAGCGTGGTCTCGATGCGATTTCACGTGATTCGCATGGCACTTCCATATTTCTTCGAGCATTGAGGGAGGCATGGGGTAG
ATGTGTTTGCCGCCCGATTGCTCTTAGGACCGCGCTCGAACGGCGCGGGTCCTTTCTCGTGCAGAGGAGGAGAATGTGAAAGAGACGACAGAGCATATTCCAAA
ACCAAGGTAAAACAGCAAGAGGAAACTAGAGATTCATCATGATTCTGACGAACACAAGTCACAAATCATCAAGCCTGTGGAAGAACATGCAAGTCGGATTGGCA
TCGAGGGATCCACGTAGTACTACGTGCATGGTGTGGCCCAGCGCCATAAAGGCGCTAGCTAGCTAGGCAGGGCCATGGCATACTGGCATGGCGGACGACGGCG
ACCGGACACGAGCCGGCGCCGGAAGAGCGTGGCCCGTACACCACCCCTTGCCTCCTCCACGTGTACCTCCTGCCGGCTTTCCCTCTTATCCCTCCACGGATCGTC
CACGTAGACCTGGCCCCTCCGCCCCTCGAGAGCAGCCACGGAGCCGCGCACACGTGTCCCCACCCGCCCACGGCCCGCGCCGAACGCCAAACGCGGAAGCGA
TTCCGGCCCGCCCGTCCGCCCGACTCGTGTAGCGCGGCCGCGTGCCCCAGCTCGCTGCCGCCCGGTGGACGCGCCCGCGCCTCGACACGCGTCCCCGCGGCAC
GTACCGGCCACGCGCGGGCGTCACGTGGAGCCGCGCCCGACGGCCCCCGCGCCATCGCACAACTGTACGGGGCGGCCGGCGCGTGGCACGTCGAGCCCCCTC

CCCCCCCGCCCCCCTCACATTTATATGGAGGCGCCACTTGCTCGCTGC

> *TaPP2C-a10* (*TaPP2C66*) promoter

AGAAATAACTTGTTGATGGGAGATTAATATGATGGAGAATAGACCCGGGGGCCATAGGTTTCACTAGTGGCTTCTCTCGAGAGCATAAGTATTACGGTGGGTGAA

CAAATTACTGTTGAGCAATTGACAGAATTGAGCATAGTTATGAGAATATCTGGGTATGATCATGTATATAGGCATCACGTCCGAGACAAGTAGACCGACTCCTGCC

TGAATCTACTACTATTACTCCACATATCGACCGCTATCCAGCATGCATCTAGAGTATTAAGTTCATAAGAACAGAGTAACGCTTTAAGTAAGATGACATGATGTAGA

GGGATAAACTCATGCAATATGATATAAACCCCATCTTGTTATCCTCGATGGCAACAATACAATACGTGCCTTGCTGCCCCTACTGTCACTGGGAAAGGACACCGCA

AGATTGAACCCAAAGCTAAGCACTTCTCCCATTGCAAGAAAGATCAATCTAGTAGGCCAAACCAAACTGATAATTCGAAGAGACTTGCAAAGATAACCAATCATA

CATAAAAGAATTCAGAGAAGATTCAAATATTGTTCATAGATAAACTTGATCATAAACCCACAATTCATCGGTCTCAACAAACACACCGCAAAAGAAGATTACATCG

AATAGATCTCCACAAGAGAGGGGGAGAACATTGTATTGAGATCCAAAAAGAGAGAAGAAGCCATCTAGCCAATAACTATGGACCCGAAGGTCTGAGGTAAACTA

CTCACACATCATCGGAGAGGCTATGGTGTTGATGTAGAAGCCCTCCGTGATCGATGCCCCCTCCGGTGGAGCTCCGGAAAAGGCCCCAAGATGGGATCTCACGG

GTACAGAAGGTTGCGGCAGTGGAATTAGGTTTTTGGCTCTGTATCTGGTAGTTTGGGGGTACGTAGGTATATATAGGAGGAAGAAGTACGTCGGTGGAGCAACGT

GGGGCCCACGAGGGTGAAGGGCGCGCCCCCCTACCTCGTGCCCTCCTGGTTGATGTTTTGACGTAGGGTCCAAGTCCTCTGGATCACGTTCGTTCCGAAAATCAC

GTTCCCGAAGGTTTCATTCCGTTTGGACTCCGTTTGATATTTTTTTCTACGAAACTCTGAAATAGGCAAAAAAAACAGCTATTCTGGGCTGGGCCTCCGGTTAATAG

GTTAGTCCCAAAAATAATATAAAAGTGTATAATAAAGCCCATTAATGTCCAAAACAGAATATAATATAGCATGGAACAATCAAAAATTATAATTGGCTCCCACATG

TTCAAGTAGTAACAGCTACACTACTCTAATGCAAGCAGTAGAGAGAAAGAGTAGGCGGTATCGGGATGATCAAGGGGGTTTTGCTTGTTGTTGGATTCCGCCTAG

GAGACGAGACACAATCTCCTCAAAATTATTTTTTTTTAGGATCCACGATCTTCTCAATCGGACGGTACGGCTCGGAGAGACTGAAAAGCGCAGCTGCACTTTTAAG

TAGTATGGAAAAAAGCATTTCAAAATTTGGGCCGTCGGGGTGACTACTACTTGGAGAGGGGTGCCCAGGAAACGATTGAGGGTTCTCCAGAAAAAAAAAGGGAAA

CGATTGAGGGGAGGGGAGGGGAGGGGAGGCGGCGCAGCCAACCACGCAGGCATTGGCGGGTGGTCCGGGTCGCATGACACGTGTCGCCACGTCGCCCCGCAC

GTGGGCCCCGACGGGCATCCGACGTGACGCGAGACAGAGGAACACAAACCCACTTCCTCCTCCTCCTCTCTTCTTCCTCCTCCCTTTGAACCAACCCAACCCAAC

CAGAGGAGCTCTCTCTCTCTTTATAGCAATAGCAATAGCTATAGCCATAGGTAGCATAGCAGTAACCAGAGAGAGACACACTCTCACTCATGGGATTCGCTTCCGC

TCTGCTGCTGCTTGTCCGGTACCCGCCGCGCGCCTCCTGTTCCTCCTCTTTTTCTTCCACCCCCGTGCCCGTGTGAGTGTGACCGGACCGCCCGCCTCATCCATCC
